# Efficient Diethylzinc/Gallic Acid and Diethylzinc/Gallic Acid Ester Catalytic Systems for the Ring-Opening Polymerization of *rac*-Lactide

**DOI:** 10.3390/molecules201219815

**Published:** 2015-12-08

**Authors:** Karolina Żółtowska, Urszula Piotrowska, Ewa Oledzka, Marcin Sobczak

**Affiliations:** 1 Department of Inorganic and Analytical Chemistry, Faculty of Pharmacy and Division of Laboratory Medicine, Medical University of Warsaw, 1 Banacha St., Warsaw 02-097, Poland; karolina_zoltowska@o2.pl (K.Ż.); piotrowska_urszula@wp.pl (U.P.); eoledzka@wp.pl (E.O.); 2 Department of Organic Chemistry, Faculty of Materials Science and Design, Kazimierz Pulaski University of Technology and Humanities in Radom, Ul. Chrobrego 27, Radom 26-600, Poland

**Keywords:** biomedical polymers, polylactide, ring-opening polymerization, zinc-based catalysts, gallic acid, propyl gallate

## Abstract

Polylactide (PLA) represents one of the most promising biomedical polymers due to its biodegradability, bioresorbability and good biocompatibility. This work highlights the synthesis and characterization of PLAs using novel diethylzinc/gallic acid (ZnEt_2_/GAc) and diethylzinc/propyl gallate (ZnEt_2_/PGAc) catalytic systems that are safe for human body. The results of the ring-opening polymerization (ROP) of *rac*-lactide (*rac*-LA) in the presence of zinc-based catalytic systems have shown that, depending on the reaction conditions, “predominantly isotactic”, disyndiotactic or atactic PLA can be obtained. Therefore, the controlled and stereoselective ROP of *rac*-LA is discussed in detail in this paper.

## 1. Introduction

Polymeric biomaterials (aliphatic polyesters, polyanhydrides, polyethers, polyamides, polyorthoesters or polyurethanes) signify one of the most interesting fields in current material chemistry. Among these materials, polylactide (PLA) is probably the most important biomedical polymer [1] and has previously been applied to the production of cell scaffolds, drug delivery systems (DDSs), sutures in tissue engineering and prostheses for tissue replacements [2,3,4,5,6,7,8].

Two methods for PLA preparation are commonly known: the polycondensation of lactic acid and the ring-opening polymerization (ROP) of lactide (LA) [1]. The polycondensation process is hampered by the typical limitations of step polymerization, whereas ROP of LA can be initiated by metal complexes and organic compounds or enzymes, both with and without alcohol [1,9,10,11,12].

Metal complexes are desirable because they can give rise to controlled polymerizations and can therefore yield materials with a well-defined number-average molecular weight (*M_n_*), as well as a narrow polydispersity index (*PD*) [1,13]. These initiators are metal alkoxide or amide coordination compounds (sometimes formed *in situ*), which are particularly useful because of their selectivity, rate and lack of side reactions. However, metal residues are undesirable for medical or pharmaceutical applications and in these cases, a low toxicity organocatalytic or enzyme catalytic systems are favorable [1].

There are two primary mechanisms for the ROP of LA: the coordination insertion mechanism for metal complexes and the activated monomer mechanism for organo/cationic initiators [1,13]. The key initiator or catalyst parameters are polymerization control, rate and stereocontrol. Stereocontrol is an important parameter, because the PLA’s tacticity influences its properties (e.g., isotactic PLA is crystalline, whereas atactic PLA is amorphous). PLA tacticity is dependent on both the type of LA and the selected initiator or catalyst [14,15,16].

During initiator selection, the biocompatibility and toxicity of the initiator or catalytic system are important issues, especially in the case of medical or pharmaceutical applications. In general, the metal-based initiators or catalysts remain in the macromolecule and during degradation, are likely to be converted into an oxide or hydroxide. For example, some Sn-, Zn- or Zr-based initiator/catalyst systems are generally considered non-toxic [1,13].

The development of reproducible and efficient DDS requires fine tailoring of the properties of the applied PLA. The microstructure of PLA (isotactic, syndiotactic, heterotactic and atactic) influences the kinetics of the biodegradation process [1,13,14,15,16].

Zinc compounds are attractive catalytic systems because they combine high activity with relatively low toxicity [1,17,18,19,20,21,22,23,24,25,26,27,28,29,30,31,32,33,34,35,36,37]. The carboxylates, halides, amino acid salts, alkoxides, phenoxy-diamine, bis(phenoxy diamine), phenoxy-imine, phenoxy imine amine, guanidinate, bis(phenoxy), calixarene and amino bis(pyrazolyl) complexes of zinc as initiators have been investigated [19,20,21,30,31,32,33,38,39]. Furthermore, the oxides have been used in ROP of *rac*-LA as heterogeneous catalysts [1,2].

In our recent study, we found catalytic systems composed of diethylzinc/gallic acid (ZnEt_2_/GAc) and diethylzinc/propyl gallate (ZnEt_2_/PGAc), synthesized for the first time, to be quite effective in the ROP of ε-caprolactone (CL). Polymerization in bulk at 40–80 °C produced poly(ε-caprolactone) (PCL) with a high yield (*ca.* 100% in some cases). Most importantly, when the ROP of CL was carried out in the presence of ZnEt_2_/PGAc catalytic system at 40–60 °C within 48 h or at 80 °C within 6 h, no macrocyclic products were formed [40].

However, the ring-opening homopolymerization of *rac*-LA alongside the application of the above-mentioned Zn-catalytic systems has not previously been studied. Therefore, in this work, the effects of temperature, reaction time and Zn-catalytic system dosage on the ROP of *rac*-LA were examined in detail. We believe that the produced PLAs, which had a well-defined microstructure, can be practically applied as “long”, “medium” or “short term” DDSs.

## 2. Results and Discussion

**Scheme 1:**
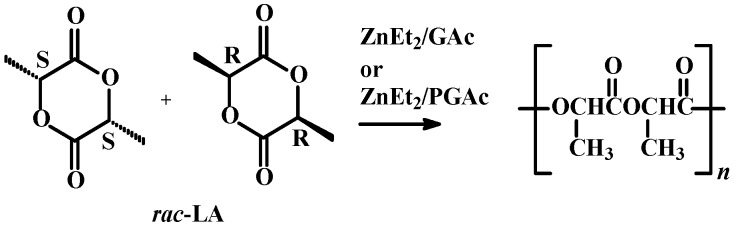
ROP of *rac*-LA in the presence of zinc-based catalytic systems.

Catalytic systems were obtained in the reaction of ZnEt_2_ with natural GAc (or PGAc) at a molar ratio of 3:1. *rac*-LA polymerizations in the presence of ZnEt_2_/GAc or ZnEt_2_/PGAc catalytic systems were carried out at zinc to monomer molar ratio of 1:50 or 1:100 at 40–80 °C (Scheme 1, Table 1, Table 2, Table 3 and Table 4). Toluene, tetrahydrofuran or dichloromethane were used as a reaction medium. The effects of the reaction medium, temperature and reaction time on the monomer conversion, product molecular weight, as well as the microstructure of the synthesized polyesters were investigated.

We found that ROP of *rac*-LA produced PLAs terminated with hydroxyl chain end groups under these conditions. The chemical structures of the obtained PLAs were confirmed by ^1^H- or ^13^C-NMR and FT-IR studies (see the Experimental Section). The molecular weight and polydispersity of the synthesized polyesters were also determined.

**Table 1:** Ring-opening polymerization of *rac*-LA in toluene in the presence of ZnEt_2_/GAc catalytic system.

Entry	Molar Ratio [Zn]/[*rac*-LA]_0_	Temp. (°C)	Time (h)	Yield ^a^ (%)	Conv. ^b^ (%)	*M_n_* ^c^ (Da)	*PD* ^c^	*MC* ^d^ (%)	*M_v_* ^e^ (Da)	*M_n_* ^f^ (Da)	*p*_2_	*L_i_*	*T*
PLA 1	1/50	40	16	36	39	2500	1.26	3	2800	2700	0.70	2.86	0
PLA 2	1/50	40	48	44	48	3200	1.48	9	3400	2900	-	-	0.22
PLA 3	1/50	60	16	35	40	2000	1.18	3	2400	2100	-	-	0.19
PLA 4	1/50	60	24	43	47	3000	1.54	10	3300	3200	-	-	0.22
PLA 5	1/50	60	48	48	52	3300	2.71	22	3400	3100	-	-	0.57
PLA 6	1/50	80	48	58	64	4000	3.39	33	4000	3700	-	-	0.85
PLA 7	1/100	40	16	28	32	2100	1.49	6	2500	2300	-	-	0.08
PLA 8	1/100	40	48	39	43	5400	1.56	7	5700	5200	-	-	0.14
PLA 9	1/100	60	24	38	43	5600	1.63	9	5800	5300	-	-	0.17
PLA 10	1/100	60	48	43	48	6000	2.49	18	6300	6100	-	-	0.47
PLA 11	1/100	80	6	37	41	5200	2.32	6	5500	5000	-	-	0.36
PLA 12	1/100	80	16	44	47	5900	2.48	17	6200	5800	-	-	0.41
PLA 13	1/100	80	24	47	52	6600	2.67	30	6600	6200	-	-	0.49
PLA 14	1/100	80	48	52	57	6800	3.21	39	7100	6400	-	-	0.76

^a^ calculated by the weight method; ^b^ calculated from ^1^H-NMR analysis (spectra of a crude reaction mixture; the conversion has been calculated by the integration of the characteristic signal of the monomer (δ = 5.03 ppm) and the polymer chain (ranged from δ = 5.13 to 5.18 ppm)); ^c^ determined by GPC; *M_n_* corrected by a factor of *ca.* 0.58 [41]; ^d^ *MC* (macrocyclic content) determined by MALDI TOF MS; ^e^ determined by viscosity method (K = 2.21 × 10^−^^4^ dL/g and α = 0.77) [42,43,44]; ^f^ determined by ^1^H-NMR; *p*_2_—coefficient of stereoselectivity calculated from the equation presented in [45]; *T*—transesterification coefficient [15]; *L_i_* = 2/*p_i_*—average length of lactyl units [46].

**Table 2:** Ring-opening polymerization of *rac*-LA in tetrahydrofuran and dichloromethane in the presence of ZnEt_2_/GAc catalytic system.

Entry	Molar Ratio [Zn]_0_/[*rac*-LA]_0_	Medium	Temp. (°C)	Time (h)	Yield ^a^ (%)	Conv. ^b^ (%)	*M_n_* ^c^ (Da)	*PD* ^c^	*MC* ^d^ (%)	*M_v_* ^e^ (Da)	*M_n_* ^f^ (Da)	*p*_2_	*L_i_*	*T*
PLA 15	1/50	THF	40	16	23	26	1600	1.29	6	1800	1300	0.63	3.17	0
PLA 16	1/50	THF	40	48	37	41	2500	2.25	13	2700	2100	-	-	0.33
PLA 17	1/50	THF	60	48	43	47	2900	3.08	29	3200	2600	-	-	0.64
PLA 18	1/100	THF	40	48	32	36	4500	2.37	11	4700	4300	-	-	0.26
PLA 19	1/100	THF	60	48	35	38	4700	2.91	24	4900	3800	-	-	0.59
PLA 20	1/50	CH_2_Cl_2_	40	24	traces	traces	-	-	-	-	-	-	-	-
PLA 21	1/50	CH_2_Cl_2_	40	48	21	23	1300	2.86	17	1700	1200	-	-	0.42
PLA 22	1/100	CH_2_Cl_2_	40	48	16	17	2100	2.32	14	2400	2000	-	-	0.37

^a^ calculated by the weight method; ^b^ calculated from ^1^H-NMR analysis (spectra of a crude reaction mixture; the conversion has been calculated by the integration of the characteristic signal of the monomer (δ = 5.03 ppm) and the polymer chain (ranged from δ = 5.13 to 5.18 ppm)); ^c^ determined by GPC; *M_n_* corrected by a factor of *ca.* 0.58 [41]; ^d^ *MC* (macrocyclic content) determined by MALDI TOF MS; ^e^ determined by viscosity method (K = 2.21 × 10^−^^4^ dL/g and α = 0.77) [42,43,44]; ^f^ determined by ^1^H-NMR; *p*_2_—coefficient of stereoselectivity calculated from the equation presented in [45]; *T*—transesterification coefficient [15]; *L_i_* = 2/*p_i_*—average length of lactyl units [46].

**Table 3:** Ring-opening polymerization of *rac*-LA in toluene in the presence of ZnEt_2_/PGAc catalytic system.

Entry	Molar Ratio [Zn]_0_/[*rac*-LA]_0_	Temp. (°C)	Time (h)	Yield ^a^ (%)	Conv. ^b^ (%)	*M_n_* ^c^ (Da)	*PD* ^c^	*MC* ^d^ (%)	*M_v_* ^e^ [Da]	*M_n_* ^f^ [Da]	*p*_2_	*L_i_*	*T*
PLA 23	1/50	40	16	39	43	2700	1.19	2	3100	2900	0.92	2.17	0
PLA 24	1/50	40	48	61	69	4500	1.42	11	4800	4200	-	-	0.13
PLA 25	1/50	60	16	53	58	3600	1.28	3	4100	3900	0.58	3.38	0
PLA 26	1/50	60	24	59	65	4100	1.38	7	4400	4300	-	-	0.05
PLA 27	1/50	60	48	68	74	4600	2.36	13	4900	4400	-	-	0.46
PLA 28	1/50	80	48	83	91	5700	3.04	31	5900	5200	-	-	0.74
PLA 29	1/100	40	16	35	39	4800	1.18	3	5300	5200	0.90	2.22	0
PLA 30	1/100	40	48	54	61	7700	1.27	5	8100	7400	0.72	2.77	0
PLA 31	1/100	60	16	42	46	5700	1.32	6	6200	5800	0.60	3.33	0
PLA 32	1/100	60	24	56	62	7900	1.31	8	8300	7500	0.61	3.28	0
PLA 33	1/100	60	48	63	68	8700	1.89	16	8900	8500	-	-	0.38
PLA 34	1/100	80	6	54	59	7600	1.39	4	7800	7200	-	-	0.16
PLA 35	1/100	80	16	62	68	8600	1.48	16	9000	8300	-	-	0.27
PLA 36	1/100	80	24	67	73	9300	2.06	25	9500	9100	-	-	0.39
PLA 37	1/100	80	48	75	82	9900	2.47	37	10,300	9400	-	-	0.59

^a^ calculated by the weight method; ^b^ calculated from ^1^H-NMR analysis (spectra of a crude reaction mixture; the conversion has been calculated by the integration of the characteristic signal of the monomer (δ = 5.03 ppm) and the polymer chain (ranged from δ = 5.13 to 5.18 ppm)); ^c^ determined by GPC; *M_n_* corrected by a factor of *ca.* 0.58 [41]; ^d^ *MC* (macrocyclic content) determined by MALDI TOF MS; ^e^ determined by viscosity method (K = 2.21 × 10^−^^4^ dL/g and α = 0.77) [42,43,44]; ^f^ determined by ^1^H-NMR; *p*_2_—coefficient of stereoselectivity calculated from the equation presented in [45]; *T*—transesterification coefficient [15]; *L_i_* = 2/*p_i_*—average length of lactyl units [46].

**Table 4:** Ring-opening polymerization of *rac*-LA in tetrahydrofuran and dichloromethane in the presence of ZnEt_2_/PGAc catalytic system.

Entry	Molar ratio [Zn]_0_/[*rac*-LA]_0_	Medium	Temp. (°C)	Time (h)	Yield ^a^ (%)	Conv. ^b^ (%)	*M_n_* ^c^ (Da)	*PD* ^c^	*MC* ^d^ (%)	*M_v_* ^e^ [Da]	*M_n_* ^f^ [Da]	*p_2_*	*L_i_*	*T*
PLA 38	1/50	THF	40	16	29	31	2000	1.35	7	2300	1800	0.71	2.82	0
PLA 39	1/50	THF	40	48	40	46	2900	1.69	18	3300	2600	-	-	0.19
PLA 40	1/50	THF	60	48	47	51	3300	2.61	22	3700	3100	-	-	0.49
PLA 41	1/100	THF	40	48	38	41	5100	1.82	12	5700	4700	-	-	0.07
PLA 42	1/100	THF	60	48	39	44	5500	2.39	26	5600	4900	-	-	0.44
PLA 43	1/50	CH_2_Cl_2_	40	16	15	17	1200	1.74	18	1500	1100	-	-	0.27
PLA 44	1/50	CH_2_Cl_2_	40	48	36	39	2400	2.89	29	2600	2100	-	-	0.53
PLA 45	1/100	CH_2_Cl_2_	40	48	32	34	4200	1.92	23	4700	3800	-	-	0.38

^a^ calculated by the weight method; ^b^ calculated from ^1^H-NMR analysis (spectra of a crude reaction mixture; the conversion has been calculated by the integration of the characteristic signal of the monomer (δ = 5.03 ppm) and the polymer chain (ranged from δ = 5.13 to 5.18 ppm)); ^c^ determined by GPC; *M_n_* corrected by a factor of *ca* 0.58 [41]; ^d^ *MC* (macrocyclic content) determined by MALDI TOF MS; ^e^ determined by viscosity method (K = 2.21 × 10^−^^4^ dL/g and α = 0.77) [42,43,44]; ^f^ determined by ^1^H-NMR; *p_2_*—coefficient of stereoselectivity calculated from the equation presented in [45]; *T*—transesterification coefficient [15]; *L_i_* = 2/*p_i_*—average length of lactyl units [46].

As shown in Table 1, Table 2, Table 3 and Table 4, the yield of the ROP process was dependent on the *rac*-LA/catalytic system’s molar ratio, reaction medium, temperature and reaction time.

The ROP yield of the *rac*-LA process ranged from 16% to 58% for ZnEt_2_/GAc (Table 1 and Table 2) and from 15% to 83% for ZnEt_2_/PGAc catalytic systems (Table 3 and Table 4). Only in one case (**PLA** **20**, Table 2) the polymeric product was obtained in a trace amount. It was found that this type of solvent had an essential influence on the process’ yield, that is, toluene was found to be an optimum polymerization medium. For ZnEt_2_/PGAc catalytic system, the yields of PLA were in the range of 35%–83% (medium—toluene, Table 3), 29%–47% (medium—THF, Table 4) and 15%–36% (medium—CH_2_Cl_2_, Table 4). In comparison, the yields of the ROP products of *rac*-LA catalyzed by ZnEt_2_/GAc ranged from 28% to 58% (medium—toluene, Table 1), 23%–43% (medium—THF, Table 2) and 0%–21% (medium—CH_2_Cl_2_, Table 2). Moreover, the ROP yields increased when the reaction temperature was raised from 40 to 80 °C. For example, PLAs were obtained with a high yield: 61% (**PLA** **24**), 68% (**PLA** **27**) and 83% yields (**PLA** **28**), respectively (Table 3). The PLA yield tended to decrease with increasing of *rac*-LA/catalytic system molar ratio. For **PLA** **27**, **PLA** **28**, **PLA** **33** and **PLA** **37**, the corresponding yield values were 68%, 83%, 63% and 75%, respectively (Table 3). The process yield also increased with the reaction time increasing. For example, PLAs were obtained with 53% (**PLA** **25**, reaction time 16 h), 59% (**PLA** **26**, reaction time 24 h) and 68% yields (**PLA** **27**, reaction time 48 h), respectively (Table 3).

The molecular weight of PLAs was also dependent on the *rac*-LA/catalytic system molar ratio, reaction medium, temperature and reaction time (Table 1, Table 2, Table 3 and Table 4). The average molecular mass (*M_n_*) values of PLA increased when the reaction time, reaction temperature and *rac*-LA/catalytic system molar ratio were increased. The *M_n_* values of PLA determined by the GPC were in the range of 1200–9900 Da (ZnEt_2_/PGAc catalytic system, Table 3 and Table 4) and 1300–6800 Da (ZnEt_2_/GAc catalytic system, Table 1 and Table 2). When the process was carried out in the presence of a ZnEt_2_/PGAc catalytic system (where the molar ratio of catalyst to monomer was 1:100, reaction temp. 80 °C), the *M_n_* results were: 9900 Da for **PCL** **37** (reaction time 48 h), 9300 Da for **PLA** **36** (reaction time 24 h) and 8600 Da for **PLA** **35** (reaction time 16 h) (Table 3). In comparison, when the ZnEt_2_/GAc catalytic system was used, *M_n_* results were 6800 Da for **PLA** **14** (reaction time 48 h), 6600 Da for **PLA** **13** (reaction time 24 h) and 5900 Da for **PLA** **12** (reaction time 16 h), respectively (Table 1).

As was shown, the PLAs obtained in the presence of ZnEt_2_/PGAc catalytic system were generally characterized by a higher *M_n_* when compared to the PLAs synthesized in the presence of ZnEt_2_/GAc. Moreover, when ROP was carried out in toluene, the synthesized PLAs were characterized by a higher *M_n_* than that of PLAs synthesized in THF or CH_2_Cl_2_. The *M*_n_ values determined from GPC were comparable to the viscosity analysis results (*M_v_*), as well as those of *M_n_* calculated from ^1^H-NMR.

**Figure 1:**
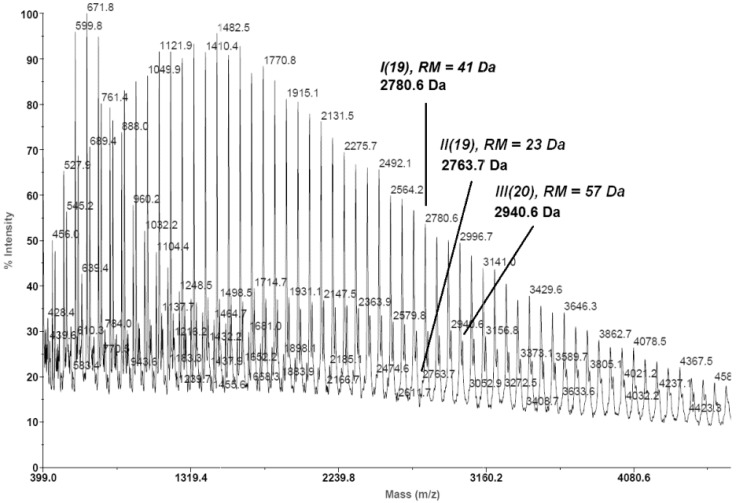
MALDI TOF MS spectrum of PLA obtained in the presence ZnEt_2_/PGAc catalytic system (**PLA** **26**).

As is known, in the MALDI-TOF MS spectra of PLA, two populations of chains can be observed (the even number and the odd number of lactyl units). An odd number of lactyl units shows that the PLA chain undergoes intra- and intermolecular transesterification. In our results, the MALDI-TOF MS spectra of the synthesized PLAs comprise two or three series of peaks (Figure 1). The primary series (***I***) corresponded to PLA macromolecule terminated with a hydroxyl group and a hydrogen atom (residual mass: *ca.* 41 Da, Na^+^ adduct). The third series of peaks (***III***) also corresponded to PLA molecules terminated with a hydroxyl group and hydrogen atom (residual mass: *ca.* 57 Da, K^+^ adduct). The second series of peaks, which had low intensity (almost unnoticeable) (***II***), corresponded to cyclic molecules (residual mass: *ca.* 23 Da, Na^+^ adduct). The content of this population was determined on the basis of the intensity ratio of the peaks for linear and cyclic PLA. As was shown, the content of cyclic products generally increased with increasing of the temperature and polymerization time. In our previous paper, we reported that when ROP of CL was carried out in the presence of ZnEt_2_/PGAc catalytic system at 40–60 °C within 48 h or at 80 °C within 6 h, macrocyclic products did not formed [40]. As shown in Table 1, Table 2, Table 3 and Table 4, trends of macrocyclization process during ROP of *rac*-LA in the presence of ZnEt_2_/PGAc were similar. The macrocyclic content (*MC*) for PLAs obtained in the presence of ZnEt_2_/PGAc catalytic system was low when compared to the *MC* of PLAs obtained in the presence of the ZnEt_2_/GAc catalytic system (Table 1, Table 2, Table 3 and Table 4). For **PLA** **23**, **PLA** **1**, **PLA** **29** and **PLA** **7**, the corresponding *MC* values were 2%, 3%, 3% and 6%, respectively.

In summary, our results clearly show that ZnEt_2_/PGAc is a more effective catalytic system for the promotion of the polymerization of *rac*-LA, compared to ZnEt_2_/GAc. The *rac*-LA monomer had almost completely been consumed in the presence of ZnEt_2_/PGAc within 48 h at 80 °C (Table 3, **PLA** **28**, conversion 91%). In comparison, the maximum conversion for ROP of *rac*-LA catalyzed by ZnEt_2_/GAc was 64% in the same reaction condition (Table 1, **PLA** **6**). The same trend was observed in our previous experiments concerning ROP of CL in the presence of ZnEt_2_/PGAc or ZnEt_2_/GAc catalytic systems. This likely demonstrates that only -OZn- active species are formed in the first case (ZnEt_2_/PGAc) whereas in the second case (ZnEt_2_/GAc), -COOZn- species are also formed [40].

It has been established that the physico-chemical, biological and biodegradation properties of PLA are dramatically dependent on the stereochemistry of PLA. Although zinc compounds have been extensively studied, these are the highest stereoselectivity, achieved by zinc-based catalysts from *rac*-LA to date [47,48,49,50,51,52,53].

The microstructure of the PLA was evaluated by homonuclear-decoupled ^1^H-NMR and ^13^C-NMR spectra. The tetrad peaks in ^1^H-NMR spectra were assigned as noted in the literature [49]. Tetrads (for the methine carbon) or hexads (for the carbonyl carbon) distribution were also observed in the ^13^C-NMR [45].

The literature notes that when an intermolecular transesterification process does not occur during polymerization, the carbonyl carbon region exhibits several lines that correspond to 11 hexads, resulting from a pair addition of enantiomers of LA. When the transesterification process occurs, new lines can be observed in the spectrum of carbonyl region as a combination of 21 hexads containing *ss* segment [45]. Moreover, when intermolecular transesterification process do not occur during polymerization, the resonanse lines due to *iss*, *sss* and *ssi* tetrads are not observed in the methine region [45,46,47,48,49,50,51,52,53,54,55,56].

The values of transesterification coefficient (*T*) were calculated from the proportion of *iss* tetrad in ^1^H- or ^13^C-NMR data using Bernoullian statistics [54].

*T* was calculated using the following equation:

(1) *T* = (*isi_0_* − *isi*)/(*isi_0_* − 0.125)

The experimental *isi* relative weight can essentially vary from 0.125 (random linkage of lactyl units) to 0.25 (Bernoullian addition of pairs). It is known that *T* values varying from 0 to 1 and in a stereoselective process the upper limit related to the *isi* tetrad relative weights is higher [11].

In our study, a racemic mixture of LA was polymerized (for the ratio of enantiomers, k = 1). It is possible to assume that the probabilities of the enantiomer addition to the growing chain terminated with the same enantiomer are equal *p*_RR/RR_ = *p*_SS/SS_ = *p_1_*. The probabilities of the enantiomer’s addition to the growing chain terminated with opposite enantiomers are equal *p*_RR/SS_ = *p*_SS/RR_ = *p_2_* (because *p*_RR/RR_ + *p*_SS/RR_ = 1 and *p*_SS/SS_ + *p*_RR/SS_ = 1) [45].

It is therefore possible to calculate the intensity values of the individual sequences:

- for tetrads

(2) (*iii*) = *p*_1_^3^ + 1.5*p*_1_^2^*p*_2_ + 0.5*p*_1_*p*_2_^2^

(3) (*iis*) = (*sii*) = 0.5*p*_1_^2^*p*_2_ + 0.5*p*_1_*p*_2_^2^

(4) (*isi*) = 0.5*p*_1_^2^*p*_2_ + *p*_1_*p*_2_^2^ + 0.5*p*_2_^3^

(5) (*sis*) = 0.5*p*_1_*p*_2_^2^ + 0.5*p*_2_^3^

- for hexads

(6) (*iiiii*) = *p*_1_^3^ + 0.5*p*_1_^2^*p*_2_

(7) (*iiiis*) = (*siiii*) = (*iisii*) = 0.5*p*_1_^2^*p*_2_

(8) (*iiisi*) = (*isiii*) = 0.5*p*_1_^2^*p*_2_ + 0.5*p*_1_*p*_2_^2^

(9) (*iisis*) = (*siiis*) = (*sisii*) = 0.5*p*_1_*p*_2_^2^

(10) (*isisi*) = 0.5*p*_1_*p*_2_^2^ + 0.5*p*_2_^3^

(11) (*sisis*) = 0.5*p*_2_^3^

The coefficient probabilities *p*_1_ and *p*_2_ were calculated from the above equations using the intensities of signals in the ^13^C-NMR spectrum [45]. In this work, the influence of the types of catalytic systems, as well as the reaction time and temperature on the chain microstructure was investigated.

**Figure 2:**
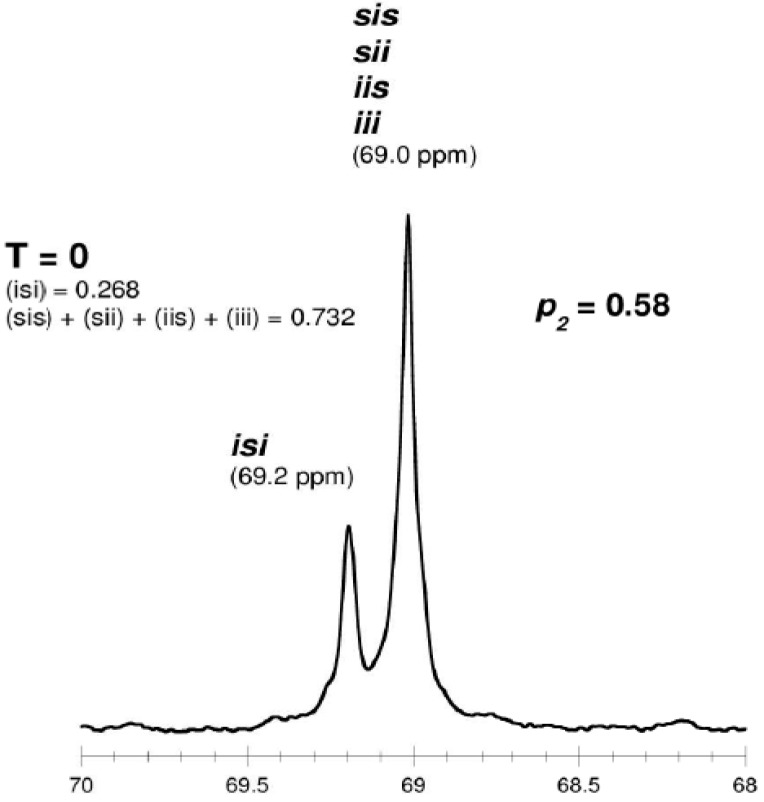
^13^C-NMR spectra of “predominantly isotactic” PLA (methine region) (*p*_2_ = 0.58, **PLA** **25**).

**Figure 3:**
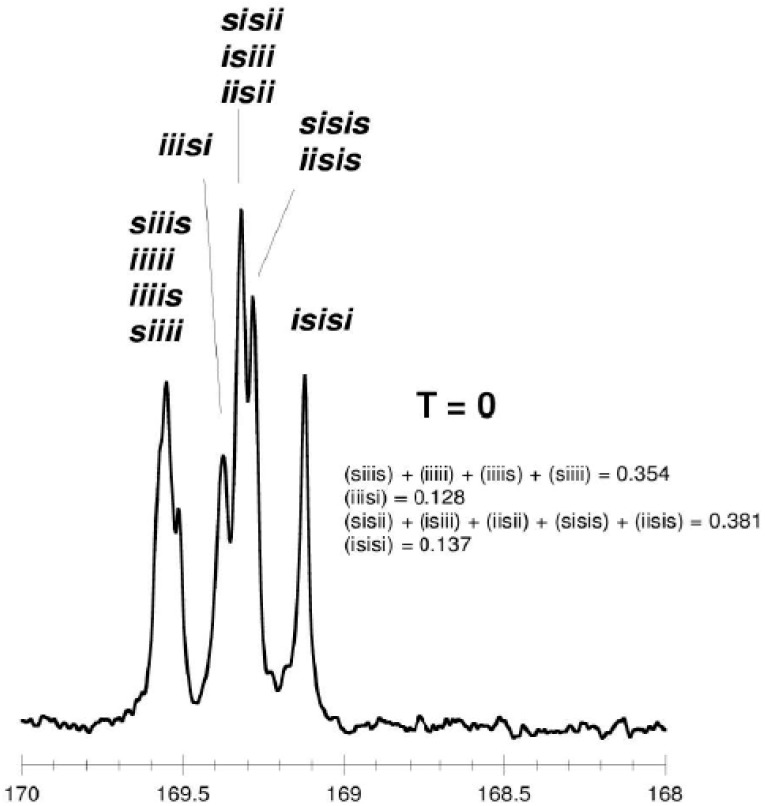
^13^C-NMR spectra of “predominantly isotactic” PLA (carbonyl region) (*p*_2_ = 0.58, **PLA** **25**).

As shown in Table 3 and Figure 2 and Figure 3, when *rac*-LA was employed using a ZnEt_2_/PGAc catalytic system (40 °C within 16–48 h or 60 °C within 16–24 h), the intermolecular transesterification process was not observed (*T* = 0). For example, for a polyester obtained in the presence of ZnEt_2_/PGAc catalytic system (60 °C within 16 h, Table 3), the calculated coefficient of stereoselectivity *p_2_* was 0.58 (**PLA** **25**), whereas the average length of lactyl units *L_i_* = 3.38 (when no *ss* sequences were present in the polymer chain, this coefficient may be defined as *p*_2_ = 2/*L_i_*, where *L_i_* is the average length of the isotactic microblocks). In our research, the “predominantly isotactic” PLA (**PLA** **25**) was obtained in the conditions stated above. As is commonly known, the ROP process of *rac*-LA enables the following to form:

isotactic PLA (*T* = 0, *p*_1_ = 1, *p*_2_ = 0) ...SSSSSS... + ...RRRRRR...

“predominantly isotactic” PLA (*T* = 0, *p*_1_ = 0.5, *p*_2_ = 0.5, *L_i_* = 4) ...SSSRRRSSSSRRR...

**Scheme 2:**
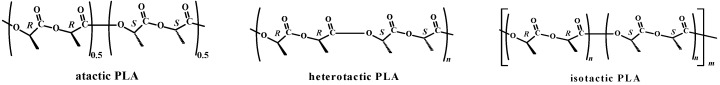
The stereostructures of PLA.

“completely disyndiotactic” (heterotactic) PLA (*T* = 0, *p*_1_ = 0, *p*_2_ = 1, *L_i_* = 2) ...SSRRSSRRSSRR...

atactic PLA (*T* = 1) ...RRSSSRSRR... (Scheme 2) [46].

**Figure 4:**
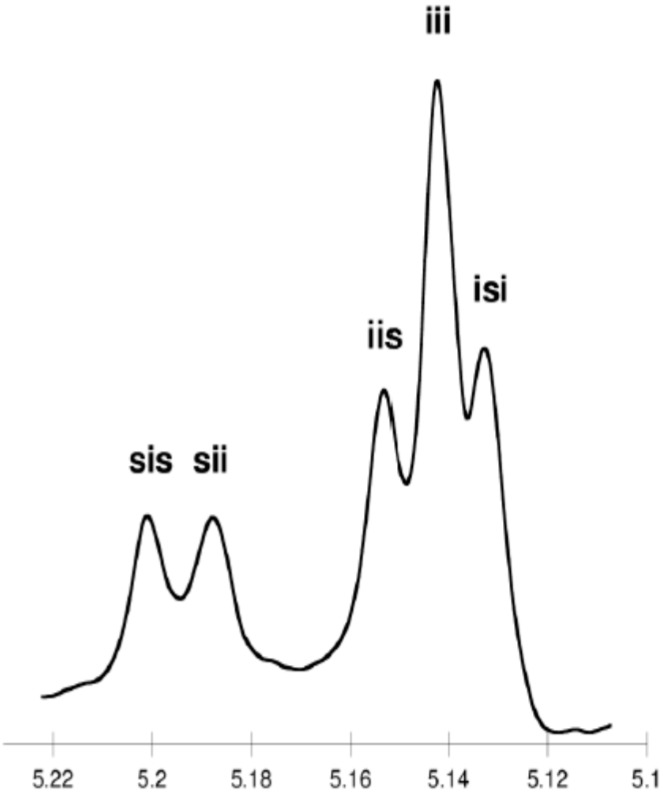
Homonuclear decoupled ^1^H-NMR spectra of the methine region of polylactide (**PLA** **14**).

**Figure 5:**
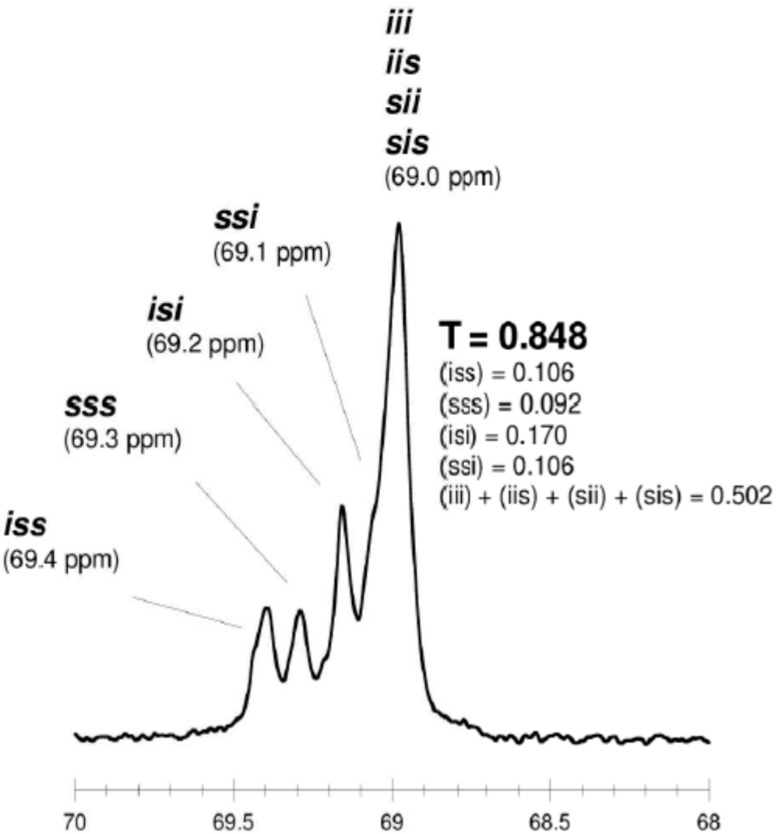
^13^C-NMR spectra of atactic PLA (methine region) (**PLA** **6**).

In the ^13^C-NMR spectra of PLA obtained in the presence of ZnEt_2_/PGAc catalytic system (40 °C within 16–48 h or 60 °C within 16–24 h), no lines due to tetrads and hexads containing the *ss* sequences were present (Figure 2 and Figure 3). In contrast, when ROP of *rac*-LA was carried out in the presence of ZnEt_2_/PGAc catalytic system at 40–80 °C within 48 h (Table 3), in the presence of ZnEt_2_/GAc catalytic system at 40 °C within 24–48 h, or at 60–80 °C within 6–48 h (Table 1), the intermolecular transesterification process was observed (*T* ≠ 0). As noted, the values of *T* generally increased with increasing of the temperature and polymerization time. For example, the *T* was 0.13 (temp. process 40 °C), 0.46 (temp. process 60 °C), 0.74 (temp. process 80 °C) for **PLA** **24**, **PLA** **27** and **PLA** **28**, respectively. Furthermore, when ROP of *rac*-LA was carried out at, e.g., 80 °C within 48 h (the presence of ZnEt_2_/GAc catalytic system), stereocontrol was not observed and an atactic material was obtained (Table 1, **PLA** **14**, Figure 4). The analysis of the intensities of the lines due to tetrads and the calculated transesterification coefficient *T* = 0.85 indicated strong intermolecular transesterification of PLA chain (e.g., **PLA** **6**), that in consequence leads to the formation of the atactic polymer (Figure 5).

**Figure 6:**
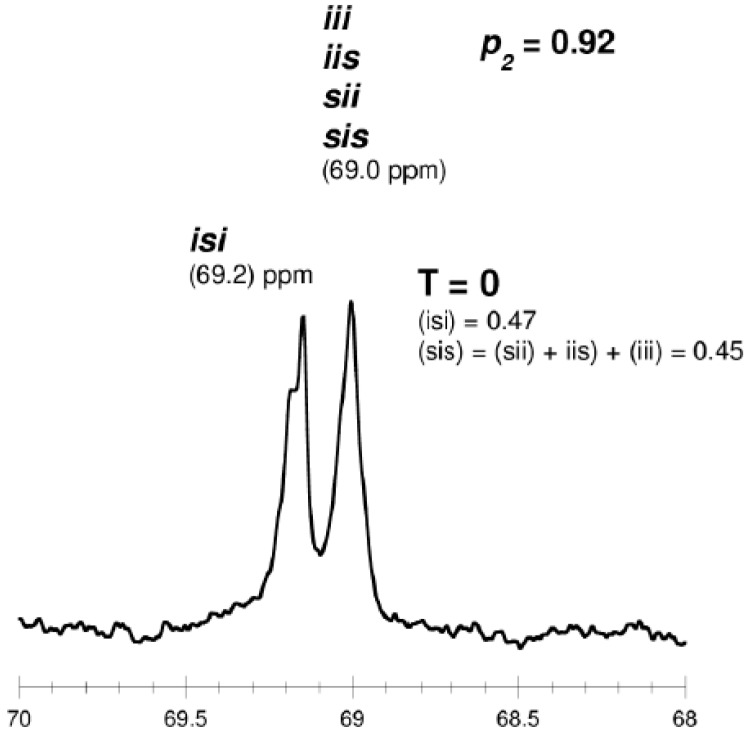
^13^C-NMR spectra of disyndiotactic PLA (methine region) (*p_2_* = 0.92, **PLA** **23**).

It is worth noting that, when ROP of *rac*-LA was carried out in the presence of a ZnEt_2_/PGAc catalytic system at 40 °C within 16 h, the microstructure of the examined polyester almost corresponded to a “completely disyndiotactic” polymer (**PLA** **23**, **PLA** **29**) (Table 3, Figure 6). In this instance, the values of *p*_2_ were roughly 0.92 and 0.90. During the reaction progress, the transesterification process tended to reduce the chain’s microstructure regularity. An analogous trend was observed by Bero when ROP of *rac*-LA was carried out in the presence of lithium tert-butoxide [55].

The results of ROP of *rac*-LA in the presence of ZnEt_2_/PGAc have also demonstrated that, depending on the conditions, “predominantly isotactic”, disyndiotactic or atactic PLA can be obtained. It is also worth noting that, when the process was carried out in the presence of a ZnEt_2_/PGAc catalytic system (40 °C within 16–48 h or 60 °C within 16–24 h), intermolecular transesterification was not observed. Generally, we can find that when the temperature and the reaction time have been increased, the microstructure of obtained PLA has been changed in the following way: disyndiotactic, “predominantly isotactic” and “completely atactic”.

**Scheme 3:**
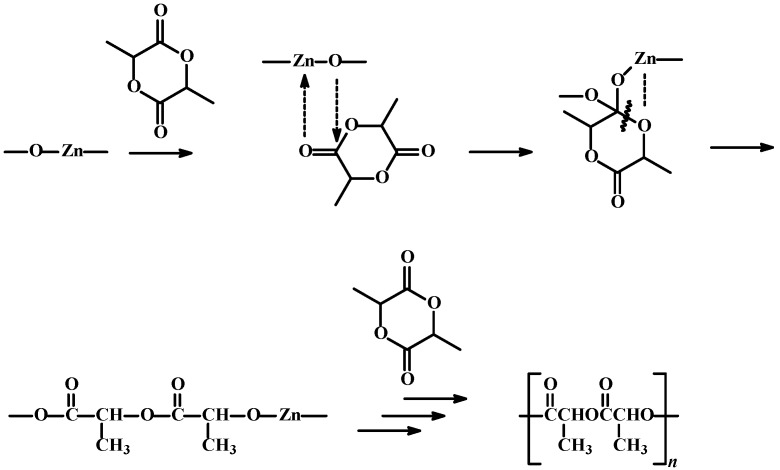
The hypothetical mechanism of ROP of *rac*-LA in the presence of ZnEt_2_/GAc and ZnEt_2_/PGAc catalytic systems.

We assume that ROP of *rac*-LA catalyzed by ZnEt_2_/GAc or ZnEt_2_/PGAc probably follows a coordination-insertion mechanism. The acidic metal center loosely binds and activates the lactide to attack by the -ZnO- group. The intermediate undergoes acyl bond cleavage of the lactide ring to generate a -ZnO- species and a growing chain end capped with an ester group (Scheme 3). However, it is difficult to obtain molecular zinc complexes (from the reaction of ZnEt_2_ with GAc or PGAc), due to the strong association tendency of the products in the reaction medium [40]. However, the relevant kinetic and mechanistic studies are underway and will be presented in our next paper.

## 3. Experimental Section

### 3.1. Materials

*rac*-Lactide (3,6-dimethyl-1,4-dioxane-2,5-dione, 99%, *rac*-LA) was purchased from Sigma-Aldrich Co. (Poznan, Poland) and further purified by crystallization from anhydrous toluene. Prior to use, the solvents (toluene, THF, CH_2_Cl_2_; Sigma-Aldrich, Co., Poznan, Poland) were dried over potassium or phosphorus pentoxide. Diethylzinc (ZnEt_2_, solution 15 wt % in toluene, Sigma-Aldrich, Co.), gallic acid (3,4,5-trihydroxybenzoic acid, GAc, 97.5%–102.5%, Sigma-Aldrich, Co.) and propyl gallate (3,4,5-trihydroxybenzoic acid propyl ester, PGAc, ≥98%, Sigma-Aldrich, Co.) were used as received from the manufacturer.

### 3.2. Synthesis of the Catalytic Systems

The diethylzinc/gallic acid (ZnEt_2_/GAc) and diethylzinc/propyl gallate (ZnEt_2_/PGAc) catalytic systems were prepared each time in an argon atmosphere at room temperature immediately before reaction. The synthesis of catalytic systems was carried out in three-necked, 100 mL round-bottomed flasks. Each glass vessel was equipped with a magnetic stirrer. The flasks contained a mixture of ZnEt_2_ (0.0177 mol) and GAc (or PGAc) (0.0059 mol) at a molar ratio of 3 to 1 and toluene as a solvent (35 mL). The reactions were carried out for about 2 h [40].

### 3.3. Synthesis of Polylactide

The ROP of *rac*-LA was carried out in triplicate, in a glass tube in the presence of ZnEt_2_/GAc or ZnEt_2_/PGAc as catalysts. The required amount of monomer and ZnEt_2_/GAc or ZnEt_2_/PGAc was placed in a 10 mL glass ampoule in an argon atmosphere. The reaction vessel was then kept standing in a thermostated oil bath at 40, 60 or 80 °C for 6 to 48 h. When the reaction time was completed, the cold reaction product was dissolved in CH_2_Cl_2_ and precipitated from distilled water with diluted hydrochloric acid (5% aqueous solution). The organic phase was separated, washed with distilled water and dried in a vacuum for 2 to 3 days.

### 3.4. Spectroscopy Data

#### 3.4.1. NMR Data

^1^H-NMR (CDCl_3_, δ, ppm): 5.10–5.25 (1H, q, -C**H**(CH_3_)-), 4.38 (1H, q, -C**H**(CH_3_)OH, end group), 1.50–1.60 (3H, d, -C**H_3_**);

^13^C-NMR (CDCl_3_, δ, ppm): 169.8 (-**C**(O)O-), 69.5 (-OC(O)**C**H(CH_3_)O-), 67.2(-OC(O)**C**H(CH_3_)OH, end group), 20.6 (-OC(O)CH(**C**H_3_)OH, end group), 17.1 (-OC(O)CH(**C**H_3_)O-);

#### 3.4.2. FT-IR Data

(KBr, cm^−1^): 2997 (υ_as_CH_3_), 2947 (υ_s_CH_3_), 2882 (υCH), 1760 (υC=O), 1452 (δ_as_CH_3_), 1348–1388 (δ_s_CH_3_), 1368–1360 (δ_1_CH+δ_s_CH_3_), 1315–1300 (δ_2_CH), 1270 (δCH + υCOC), 1215–1185 (υ_as_COC + r_as_CH_3_), 1130 (r_as_CH_3_), 1100–1090 (υ_s_COC), 1045 (υC-CH_3_), 960–950 (rCH_3_ + υCC), 875–860 (υC-COO), 760–740 (δC=O), 715–695 (γC=O), 515 (δ1C-CH_3_ + δCCO), 415 (δCCO), 350 (δ2C-CH_3_ + δCOC), 300–295 (δCOC + δ2C-CH_3_), 240 (τCC);

### 3.5. Measurements

The intrinsic viscosity of PLAs was determined in *N*,*N*-dimethylformamide (DMF) (at 30 °C) using a Stabinger Viscometer SVM 3000. The concentrations of the PLA solutions in DMF were as follow: 0.2%, 0.4%, 0.6%, 0.8% and 1%. The viscosity average molecular weight was calculated with the Mark–Houwink equation using the following constants: K = 2.21 × 10^−^^4^ dL/g and α = 0.77 [42,43,44].

Number-average molecular weight and polydispersity were determined by gel permeation chromatography (GPC). The GPC instrument (GPC Max + TDA 305, Viscotek) was equipped with Jordi DVB Mixed Bed columns (one guard and two analytical) at 30 °C in CH_2_Cl_2_ (HPLC grade, Sigma-Aldrich) and at a flow rate of 1 mL/min, with RI detection and calibration based on narrow PS standards (ReadyCal Set, Fluka). The results were processed with OmniSEC software (ver. 4.7. Houston, TX, USA).

MALDI-TOF mass spectra were performed in a linear mode using an ultrafleXtreme™ (Bruker Daltonics, Coventry, UK) mass spectrometer using a nitrogen gas laser and DCTB as a matrix. The PLA samples were dissolved in THF (5 mg/mL) and mixed with a solution of DCTB.

The polymerization products were characterized by means of ^1^H- or ^13^C-NMR (using Varian 300 MHz recorded, Palo Alto, CA, USA) in deuterated chloroform (CDCl_3_) at room temperature. FT-IR spectra (PerkinElmer, Waltham, MA, USA) were measured from KBr pellets.

## 4. Conclusions

In this study, we described for the first time the synthesis and characterization of polylactides obtained in the presence of two zinc-based catalytic systems. The biocompatible ZnEt_2_/GAc and ZnEt_2_/PGAc catalytic systems were shown to be effective for the coordination-insertion ring-opening polymerization of *rac*-lactide (*rac*-LA). Zinc catalytic systems were proven as promising catalysts not only for molecular weight control, but also for stereocontrol. It was found that when *rac*-LA was polymerized with ZnEt_2_/PGAc catalytic system (40 °C within 16–48 h or 60 °C within 16–24 h), the intermolecular transesterification process was not observed. Furthermore, “predominantly isotactic” PLA was obtained in these reaction conditions. In addition, when ROP of *rac*-LA was carried out in the presence of a ZnEt_2_/PGAc catalytic system (40 °C within 16 h), the microstructure of the examined polyester practically corresponds to “completely disyndiotactic” polymer. Efforts aimed at subsequent improvement of the stereocontrol of ROP of *rac*-LA in the presence of ZnEt_2_/GAc and ZnEt_2_/PGAc catalytic systems, as well as understanding the origin of isoselectivity and detailed reaction mechanisms, are currently underway in our laboratory.
